# Enhanced Thermoelectric Performance by Surface Engineering in SnTe-PbS Nanocomposites

**DOI:** 10.3390/ma14185416

**Published:** 2021-09-19

**Authors:** Cheng Chang, Maria Ibáñez

**Affiliations:** Am Campus 1, Institute of Science and Technology Austria, 3400 Klosterneuburg, Austria; cheng.chang@ist.ac.at

**Keywords:** thermoelectric, SnTe, grain size, carrier mobility, nanocomposites

## Abstract

Thermoelectric materials enable the direct conversion between heat and electricity. SnTe is a promising candidate due to its high charge transport performance. Here, we prepared SnTe nanocomposites by employing an aqueous method to synthetize SnTe nanoparticles (NP), followed by a unique surface treatment prior NP consolidation. This synthetic approach allowed optimizing the charge and phonon transport synergistically. The novelty of this strategy was the use of a soluble PbS molecular complex prepared using a thiol-amine solvent mixture that upon blending is adsorbed on the SnTe NP surface. Upon consolidation with spark plasma sintering, SnTe-PbS nanocomposite is formed. The presence of PbS complexes significantly compensates for the Sn vacancy and increases the average grain size of the nanocomposite, thus improving the carrier mobility. Moreover, lattice thermal conductivity is also reduced by the Pb and S-induced mass and strain fluctuation. As a result, an enhanced *ZT* of ca. 0.8 is reached at 873 K. Our finding provides a novel strategy to conduct rational surface treatment on NP-based thermoelectrics.

## 1. Introduction

Thermoelectric materials, which can directly convert heat into electricity, are promising candidates for low-grade heat exploitation [1,2,3,4,5,6,7]. The energy conversion efficiency is limited by the figure of merit *ZT*, *ZT* = *σS*^2^*T*/(*κ*_lat_ + *κ*_ele_), where *σ*, *S*, *κ*_lat_, *κ*_ele_, *T* are the electrical conductivity, Seebeck coefficient, lattice thermal conductivity, electronic thermal conductivity, and absolute temperature, respectively. To date, significant progress has been made by applying different strategies to synergistically modify charge and phonon transport, including band convergence [8], all-scale hierarchical phonon scattering [9,10], optimizing materials with intrinsically low lattice thermal conductivities [11,12], etc. These strategies are mainly built on the top-down approach utilizing melting and sintering methods, which are time- and energy-consuming.

Recently, the bottom-up assembly of solution-processed nanoparticles (NPs) has provided the possibility to design alternative nanostructured materials while utilizing mild synthesis methods and inexpensive equipment [13,14]. Most metal chalcogenides thermoelectric materials have been produced by bottom-up solution methods, such as PbQ, Bi_2_Q_3_, SnQ (Q = Te, Se, S), etc. [1,15,16]. However, their TE performance is usually inferior to their equivalents synthesized by top-down approaches. One of the main issues is the lack of facile and effective means to tune charge carrier concentration [17].

Here, we present a novel approach to optimize the charge and phonon transport simultaneously by utilizing a NP surface treatment before their consolidation. Specifically, we demonstrate the potential of our strategy for SnTe NPs. SnTe is a promising thermoelectric material with high electrical conductivity [7,18,19]. Undoped SnTe shows poor thermoelectric performance due to the low Seebeck coefficient, which derives from the excessively high carrier concentration of >10^21^ cm^−3^ and large thermal conductivity [7,20,21]. To address such problems, we employed a unique surface treatment to reduce the carrier concentration and the thermal conductivity. In particular, we modified SnTe NPs with PbS molecular complexes. The strategy allowed (i) reducing the carrier concentration due to Pb-induced vacancy compensation, (ii) enhancing mobility due to a reduction of the grain boundary density, and (iii) reducing *κ*_lat_ by Pb and S-induced mass and strain fluctuations. Overall, thanks to the PbS surface treatment, a high *ZT* of ca. 0.8 was obtained in SnTe-PbS nanocomposites at 873 K.

## 2. Materials and Methods

SnCl_2_·2H_2_O 98%, NaOH, pellets 98%, NaBH_4_, 98%, PbO, 99.99%, and N-Methylformamide (MFA, 99%) were purchased from Fisher Scientific(Austria) GmbH (Wien, Austria). Te 100%, ethylenediamine, 99% (en), 1,2-ethanedithiol ≥ 95.0% (EDT), extra dry acetone, and ethanol (99.5%) were purchased from Sigma-Aldrich (Darmstadt, Germany). All chemicals were used as received without further purification.

SnTe NPs were synthesized by the method reported by Guang Han et al. [22]. Details can be found in SI. The PbS molecular complex preparation method applied in this work was developed by R. L. Brutchey et al. [23]. The solubility of PbO in en+EDT solvent (1:10) is ca. 20–30%. Here, we dissolved 100 mg PbO with 1.1 mL en+EDT solvent (1 mL en, 0.1 mL EDT) in a N_2_-filled vial. The mixture was sonicated until complete dissolution. All the PbS molecular ink was prepared fresh before blending with SnTe NPs in MFA.

All surface treatments were performed in an inert atmosphere (N_2_). We used 5 mL MFA to disperse 0.75 g SnTe in a 20 mL vi al. SnTe with different molar amounts of PbS molecular complex was prepared (1/2/3 mol% PbO), then the mixture was vigorously stirred (800 rpm) at room temperature for 24 h. After that, the mixture was rinsed with acetone 3 times.

As-prepared SnTe-xPbS (x = 1%, 2%, and 3% PbO molecular precursors) nanocomposites were firstly annealed at 650 °C for 120 min under a slow forming gas (95% N_2_ + 5% H_2_) flow inside a tube furnace (MTI Co., Shenyang, China) with ca. 10 °C/min heating rate. Afterward, the annealed nanopowder was ground with an agate mortar and loaded into a graphite die in a nitrogen-filled glovebox. The nanopowder was then consolidated into pellet (Ø 8.6 mm × h 2 mm) under vacuum in an AGUS PECS Spark Plasma Sintering (SPS) System-Model SPS 210Sx (SUGA Co., Ltd., Hokkaido, Japan). First, the axial pressure was slowly increased to 45 MPa in 0.5 min and kept at that pressure during the sintering process. After that, the temperature was rapidly increased from room temperature to 600 °C within 6 min and slowly increased to 650 °C within 1.5 min. Then the sample was kept at 650 °C for 5 min. All consolidated pellets presented relative densities of >98% of the theoretical value.

X-ray diffraction analyses were carried out on a Bruker AXS D8 ADVANCE powder diffractometer (Bruker, Billerica, MA, USA). The morphology and element composition of as-prepared SnTe were examined by field-emission scanning electron microscopy and an energy dispersive X-ray spectrometer (EDX, Oxford, UK) on an Auriga Zeiss operated at 5.0 kV and 15.0 kV, respectively. Both the Seebeck coefficient and the electrical resistivity were simultaneously measured in an LSR-3 LINSEIS system (Linseis Messgeraete GmbH, Vielitzerstr, Germany) from room temperature to 873 K under a helium atmosphere. Room-temperature hall charge carrier concentrations (*n*_H_) and mobilities (*μ*_H_) were measured with the Van der Pauw method using a magnetic field of 0.6 T (ezHEMS, NanoMagnetics, NanoMagnetics Instruments, Ltd., Oxford, UK). An LFA 1000 Laser Flash (Linseis Messgeraete GmbH, Vielitzerstr, Germany) was used to determine the samples’ thermal diffusivities (α).

## 3. Results and Discussions

**Figure 1:**
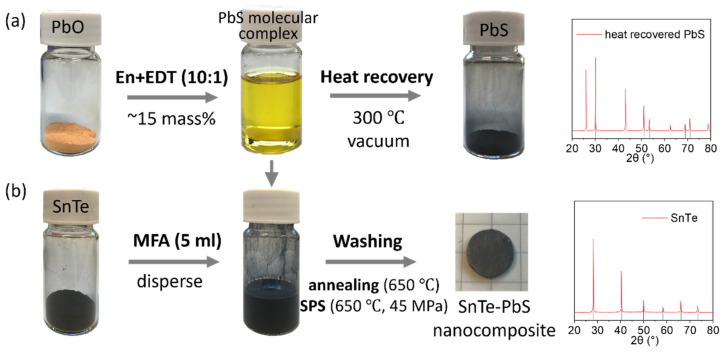
(**a**) The schematic of PbS molecular complex heat recovery and (**b**) SnTe-PbS nanocomposites assembly processing with the corresponding XRD pattern of heat recovered PbS and PbS surface-modified SnTe NPs.

The PbS molecular complex was injected into SnTe NP suspension in N-Methylformamide (MFA), and was absorbed on the NPs surface. Experiments showed that such PbS molecular complex decomposed under mild annealing (300 °C) and transformed into crystalline PbS (Figure 1a). Therefore, the PbS surface-modified SnTe NPs yielded SnTe-PbS nanocomposites (Figure 1b).

**Figure 2:**
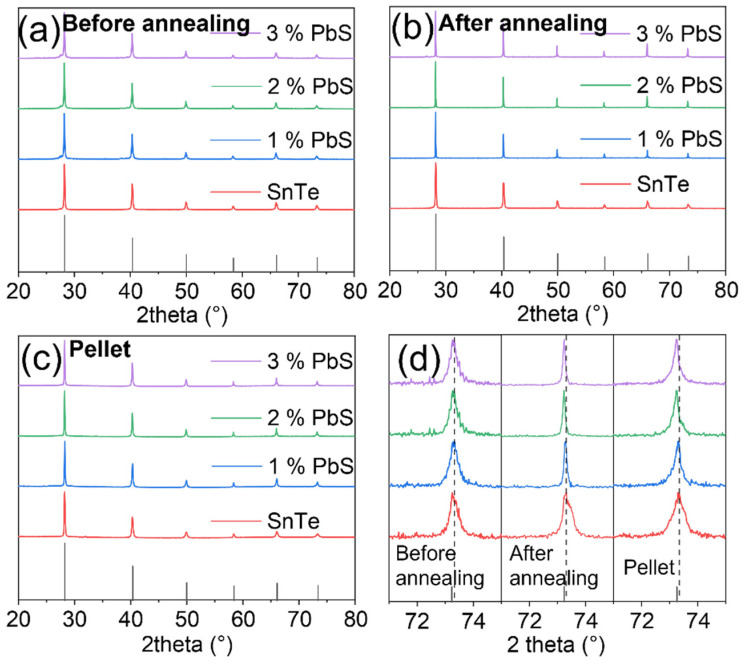
The XRD patterns of powders (**a**) before annealing, (**b**) after annealing, and (**c**) pellets. (**d**) Amplified XRD peak in the range of 71–75 °C.

Figure 2a–c shows the XRD patterns of SnTe and PbS surface-modified SnTe NPs, prepared with different content of PbS, before and after annealing, and the corresponding consolidated pellets using annealed NPs. Diffraction patterns matched to SnTe rock-salt structure without any additional peaks. No peak shift was observed either in XRD patterns before powder annealing. In contrast, after the thermal processing, we observed small peak shifts, Figure 2d. The calculated lattice parameters of pellets and the Vegard’s law line are shown in Figure S1, indicating the solid solution between SnTe and PbS.

**Figure 3:**
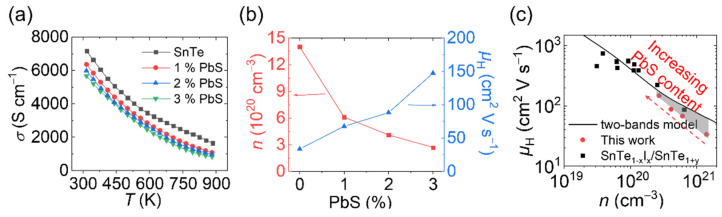
The electrical transport properties of SnTe nanomaterial and SnTe-PbS nanocomposites. (**a**) The electrical conductivity; (**b**) the carrier concentration and carrier mobility; (**c**) the carrier mobility as a function of carrier concentration.

To evaluate the effect of PbS molecular complex on the TE performance of SnTe, we analyzed the electrical and thermal transport properties. As shown in Figure 3a, the electrical conductivity of SnTe and SnTe-PbS nanocomposites showed metallic behavior, with the electrical conductivity decreasing as the temperature increased. Compared with pristine SnTe, the room temperature electrical conductivity decreased from ca. 7200 S cm^−1^ to ca. 6000 S cm^−1^ as the amount of PbS in the composite increased up to 3%. Such a decrease in electrical conductivity with the increasing amount of PbS was maintained through the whole temperature range studied. To investigate the origin of the electrical conductivity reduction, room temperature hall measurements were performed, Figure 3b. The carrier concentration was 1.4 × 10^21^ cm^−3^ in the SnTe nanomaterial and decreased to 2.7 × 10^20^ cm^−3^ in the SnTe-3% PbS nanocomposite. The decreased carrier concentration in SnTe-PbS derived from the Pb-induced vacancy compensation. It is well-known that large content of Sn vacancies results in excessively high carrier concentration in pristine SnTe. The large amounts of Sn vacancies in SnTe were due to their negative formation energy [24]. In comparison, the formation energy of Pb vacancies was much higher in PbTe. Accordingly, Pb was expected to fill the Sn vacancy by forming a solid solution [6,24]. 

Figure 3b shows the carrier mobility as a function of nominal PbS amount. In undoped SnTe, the room temperature carrier mobility was only 34 cm^2^ V s^−1^. In contrast, the hall carrier mobility in SnTe prepared by melting method was ca. 400 cm^2^ V s^−1^ [25]. We attribute the low carrier mobility to the point defect scattering from the intrinsic Sn vacancies or possible impurities (e.g., Na, C, H, O) introduced during the synthesis, the intense electron-electron scattering from the high carrier concentration, and the strong grain boundary scattering from the small grain size [26]. Strikingly, the carrier mobility improved significantly in SnTe-PbS nanocomposites, increasing to 150 cm^2^ V s^−1^ for 3% PbS content. The remarkable carrier mobility enhancement cannot be solely attributed to the reduced carrier concentration and thus reduced electron-electron scattering. In Figure 3c, the relationship between carrier mobility and carrier concentration is compared with the one derived from the two-bands model using a Kane band (SKB) for the light and a parabolic (SPB) for the heavy valence band [27]. The non-negligible deviation between the experimental data and the calculated model curve in this work, grey areas in Figure 3c, especially for the pristine SnTe, indicates that other factors played a role in the carrier mobility tuning (The SnTe_1−x_I_x_ and SnTe_1+y_ data were taken from reference [27]). After investigating the materials’ microstructure, we found that the surface treatment promoted grain growth during the consolidation. This phenomenon explains the abnormal carrier mobility trend, where grain boundary scattering is significantly reduced due to the lower grain boundary density.

**Figure 4:**
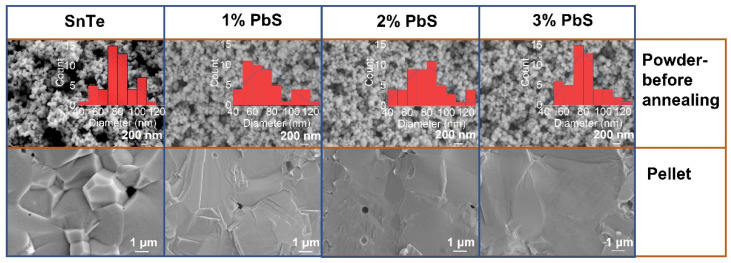
The SEM images of powders before annealing; and pellets.

The SEM images of all NPs and the corresponding consolidated pellets are shown in Figure 4 (The SEM images of powders after annealing can be found in Figure S2). The as-synthesized SnTe NPs showed irregular spherical shape with a dimension of ca. 80 nm. No apparent shape and size changes were observed after the thiol-amine surface treatment. However, the NP morphology of each sample changed dramatically after the thermal processing. The grains in SnTe-PbS nanocomposites were much larger than the bare SnTe with a dimension of >10 μm, with larger grains as we increased the content of PbS.

**Figure 5:**
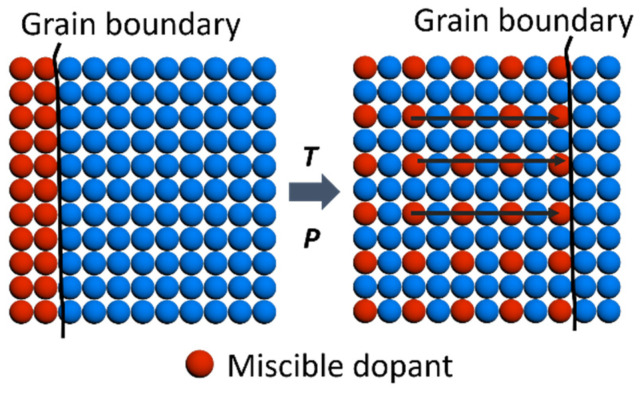
A schematic of diffusion-induced grain boundary migration.

Grain growth during the pressure-assisted sintering through spark plasma sintering (SPS) is associated with a diffusion-induced grain boundary [28,29]. The high temperature and pressure promoted the formation of a solid solution through PbS migration from the surface to the inner grain [30,31], as illustrated in Figure 5. The EDX mapping of the pellets in Figure S3 shows Pb homogeneously distributed in the SnTe, confirming the atomic diffusion process. This phenomenon happened because PbS and SnTe can form a complete solid solution [25]. As a result, the grain boundary moved along the atomic diffusion, leading to enhanced grain growth. Therefore, all SnTe-PbS nanocomposites have an average larger grain size than bare SnTe pellets [32]. Correspondingly, the larger grain sizes reduce the grain boundary density, decreasing electron grain boundary scattering and leading to higher carrier mobility in SnTe-PbS nanocomposites.

**Figure 6:**
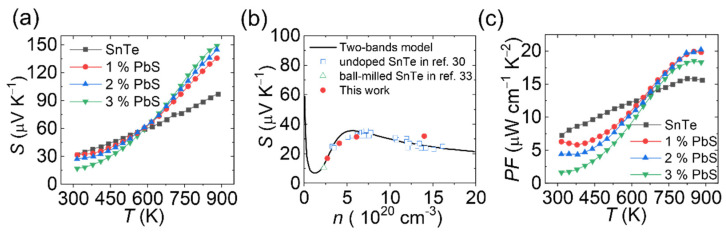
(**a**) The Seebeck coefficient as a function of temperature; (**b**) the Pisarenko relationship at room temperature; (**c**) the power factor as a function of temperature.

Figure 6a shows the temperature-dependent Seebeck coefficients. The Seebeck coefficients showed positive values for all the materials explored in the whole temperature range, indicating the p-type nature of the material associated with the intrinsic Sn vacancies. After PbS addition, the room temperature Seebeck coefficients decreased with incrementing PbS content. However, above 600 K, the tendency was inverted and the Seebeck coefficients increased in value for the material with higher content of PbS. A similar phenomenon was reported in Sn_1+x_Te, where excess Sn was introduced to compensate for Sn vacancies [26].

Figure 6b shows the room temperature Seebeck coefficient behavior as a function of carrier concentration (the undoped and ball-milled SnTe data are taken from reference [33,34]). The carrier concentration-dependent Seebeck coefficient was the opposite of the expected behavior for a p-type semiconductor with the single parabolic band, where the Seebeck coefficient was reversely proportional to the carrier concentration. A sharp Seebeck coefficient upturn was detected in the carrier concentration range of 1.2 × 10^20^ cm^−3^ to 5.5 × 10^20^ cm^−3^. This anomalous Seebeck coefficient behavior was related to the unique character of the two non-degenerate valence bands in SnTe, the light valence band and the heavy valence band. Zhang et al. calculated the Pisarenko relationship applying the two-bands model [33]. When the carrier concentration was high, the fermi level in SnTe was pushed down, crossing both the light and heavy valence bands. In this case, both valence bands contributed to the charge carrier transport, leading to large effective mass and Seebeck coefficient. When the carrier concentration decreased, the Fermi level gradually lifted away from the heavy valence band. As a result, the effective mass and Seebeck coefficient decreased with lower carrier concentration. In this work, the carrier concentration coincidently lay in this heavy valence transition region.

Notably, the Seebeck coefficient of SnTe nanomaterial was above the two-band Pisarenko line and was also higher than SnTe references with similar carrier concentrations. Considering the high grain boundary density in undoped SnTe, we speculate that energy barrier effects enhanced the Seebeck coefficient [35]. With increasing temperature, the detrimental effect of single valence band transport on the Seebeck coefficient was offset. Because of thermal activation, charge carriers have high enough energy to occupy the heavy valence band [9,36], which led to the Seebeck coefficient at 873 K increasing significantly from 97 μV K^−1^ to 150 μV K^−1^ with the rising PbS amount. Benefitting from the enhanced Seebeck coefficient and moderate electrical conductivity at high temperatures, the SnTe-PbS nanocomposites had much higher power factors than bare SnTe, with maximum values of ca. 20 μW cm^−1^ K^−1^ at 873 K, Figure 6c.

**Figure 7:**
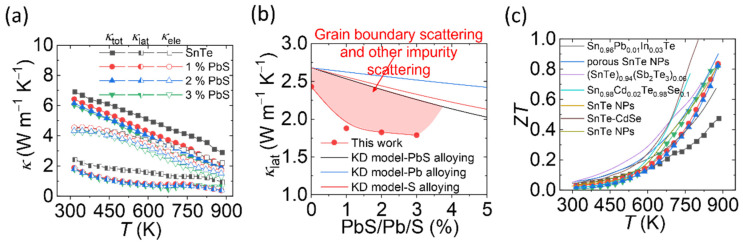
(**a**) The thermal conductivity as a function of temperature; (**b**) the lattice thermal conductivity as a function of Pb content; (**c**) *ZT* as a function of temperature. The SnTe references are listed in SI.

The temperature-dependent thermal conductivities (*κ*_tot_, *κ*_lat_, *κ*_ele_) for SnTe-PbS nanocomposites are shown in Figure 7a. The heat capacity and specific heat can be found in Figures S4 and S5). The lattice and electronic thermal conductivity can be obtained by the Wiedemann-Franz relationship:

(1) $$\kappa_{\mathrm{lat}}=\kappa_{\mathrm{tot}}-\kappa_{\mathrm{ele}}=\kappa_{\mathrm{tot}}-L\sigma T$$

The Lorenz number *L* is estimated by the Seebeck coefficient data and the reduced chemical potential using a single parabolic band model with acoustic phonon scattering, Figure S6. With increasing PbS content, *κ*_tot_ decreases gradually because of the reduction in both *κ*_lat_ and *κ*_ele_. The decreased *κ*_ele_ comes from the reduced electrical conductivity. *κ*_lat_ shows strikingly low values with the lowest being ca. 0.37 W m^−1^ K^−1^ at 873 K, which is even lower than the theoretically minimum *κ*_min_ of 0.5 W m^−1^ K^−1^ for SnTe calculated using the disordered crystal model [37].

(2) $$\kappa_{\min}=\frac{\pi }{4}k_{B}V^{-\frac{2}{3}}v$$

where *V* is the unit cell volume, *k*_B_ is the Boltzmann constant, and *v* is the sound velocity (ca. 1800 m s^−1^ for SnTe [38]).

To get a deep insight into the origin of low *κ*_lat_ in the SnTe-PbS system, we made a comparison between the experimental data and the Klemens-Drabble (KD) model [25]. The detailed calculations are shown in the SI. In the KD model, the *κ*_lat_ reduction of the doped or alloyed crystal depends on the disorder parameter Γ, depending on mass and strain fluctuations.

(3) $$\Gamma =x(1-x)\left[\left(\frac{\Delta M}{M}\right)+\varepsilon \left(\frac{\Delta a}{a}\right)\right]$$

where *x* is the dopant content in a binary system. *ε* is a phenomenological parameter related to the Grüneisen parameter, *M* and *a* are the molar mass and lattice constant of the alloy, and Δ*M* and Δ*a* are the differences in mass and lattice constant between the two constituents. The higher Γ is, the lower *κ*_lat_ will be. The calculated *κ*_lat_ as a function of Pb amount is shown as the solid black line in Figure 7b. It is clear that the experimental data lie well below the calculated values, indicating additional phonon scattering factors that contribute to the further *κ*_lat_ reduction. Considering the microstructure and the composition of SnTe-PbS nanocomposites, we speculated that the grain boundary scattering, possible formation of PbS nanoprecipitates, and other impurity scattering may be responsible for the *κ*_lat_ reduction.

Combining the enhanced power factor and the significantly reduced thermal conductivity allowed achieving a remarkable *ZT* enhancement with respect to bare SnTe at high temperatures, increasing from 0.47 to 0.82 at 873 K, Figure 7c. Compared with other bottom-up assembled SnTe [14], SnTe-PbS nanocomposites revealed moderately high *ZT* while utilizing more facile and inexpensive synthetic methods.

## 4. Conclusions

We synthetized SnTe nanoparticles in water and treated their surface with different amounts of PbS complexes. The PbS surface-treated SnTe particles were then consolidated in a bulk pellet. Thanks to such surface treatment, positive synergistic effects were achieved in both electrical and thermal transport properties, enhancing the thermoelectric performance. For one site, the ultrahigh carrier concentration was reduced by Pb-induced Sn vacancy compensation. Moreover, the formation of a solid solution with PbS promotes grain growth, hence contributing to the high carrier mobility. Finally, *κ*_lat_ was significantly reduced because of the Pb- and S-induced mass and strain fluctuation and grain boundary scattering. As a result, a moderate-high *ZT* of 0.82 was achieved at 873 K. Our work provides a new simple and versatile approach to produce bottom-up processed thermoelectric materials through surface treatments.

## Data Availability

The data presented in this study are available on a reasonable request from the corresponding author.

## Supplementary Materials

The following are available online at https://www.mdpi.com/article/10.3390/ma14185416/s1, Figure S1: The lattice parameter as a function of PbS amount. Vegard’s law is listed for comparison, Figure S2: The SEM images of powders after annealing, Figure S3: EDX mapping of SnTe-1/2/3% PbS, Figure S4: The heat capacity *C*_p_ of SnTe as a function of temperature. This figure of *C*_p_ values is taken from previous work by Zhao et al. [5] *C*_p_ in some other references are listed for comparision [6,7,8,9], Figure S5: The temperature dependent thermal diffusivity of SnTe-xPbO nanocomposites (x = 0, 1%, 2%, 3%), Figure S6: The temperature dependent Lorenz number of SnTe-xPbO nanocomposites (x = 0, 1%, 2%, 3%).
