# Supplementary material for: Enhanced Thermoelectric Performance by Surface Engineering in SnTe-PbS Nanocomposites
Source: Materials (Basel). 2021 Sep 19;14(18):5416. doi: 10.3390/ma14185416 (PMC8466123; doi:10.3390/ma14185416)
Supplement: Supplementary file 1 [file materials-14-05416-s001.zip › materials-1364820-supplementary.pdf]

SUPPORTING INFORMATION

Enhanced Thermoelectric Performance by Surface Engineering in SnTe-PbS Nanocomposites

Cheng Chang [1], and Maria Ibáñez [1,*]

[†]Institute of Science and Technology Austria, Am Campus 1, 3400 Klosterneuburg, Austria.

KEYWORDS: thermoelectric; SnTe; grain size; carrier mobility

* *E-mail:* M. Ibáñez: *mibanez@ist.ac.at*

1. SnTe nanoparticle (NP) synthesis

Large scale SnTe nanoparticles (NPs) were prepared by reacting sodium stannite (Na_2SnO_2) with sodium hydrogen telluride (NaHTe) in water following a procedure previously reported by Guang Han *et al* with slight modifications. [1]

Firstly, $NaBH_4$ (4.54 g, 120 mmol) was first dissolved in 200 ml deionized water, and then Te powder (5.104 g, 40 mmol) was quickly added into the solution. An Ar flow was introduced to prevent the air. The reaction rate between $NaBH_4$ and Te is very slow and takes ~2 h to react entirely. The suspension was stirred until the solution becomes transparent light purple, indicating the formation of NaHTe. In parallel, NaOH (16 g, 400 mmol) and $SnCl_2{\cdot}2H_2O$ (9.826 g, 40 mmol) were mixed with 200 ml of deionized water. The mixture was stirred at room temperature under Ar flow until the solution became transparent, indicating the formation of Na_2SnO_2. When NaHTe solution is ready, the Na_2SnO_2 solution was heated to its boiling point at *ca.* ~101 ºC with a condenser to assure reflux. Then the freshly prepared NaHTe solution was rapidly injected. Since NaHTe is sensitive to the air, two 100ml syringes are prepared to inject NaHTe. Upon injection, the solution color changed from transparent to black, indicating the SnTe NP formation. The mixture was heated again to 101 ºC and maintained at this temperature for 30 min. Then the mixture was cooled to room temperature with cooling water. NPs precipitated quickly when the stirring stopped. The transparent supernatant solution was carefully removed. The remaining crude was transferred into centrifuge tubes for further purification.

The SnTe NPs were washed four times with ethanol and acetone alternatively. At each step, SnTe NPs were dispersed by ethanol/ acetone first and then centrifuged at 8000 rpm for 5 min. At last, the washed NPs were dried under vacuum overnight at room temperature and kept in the glovebox for further use.

2. PbS molecular ink preparation

The PbS molecular ink preparation method applied in this work was developed by R. L. Brutchey *et al.*[2] The solubilities of Pb in en+EDT solvent (1:10) is ~20 %. Here, we dissolve 100 mg PbO with 1.1 ml en+EDT solvent (1 ml en, 0.1 ml EDT) in N_2-filled vial. The mixture was sonicated for 1 min to accelerate the dissolving processing until complete dissolution. All the PbS molecular ink were prepared fresh before blending with SnTe NPs in MFA.

3. Lorenz number estimation

An estimation of L can be made using a single parabolic band (SPB) model with acoustic phonon scattering. The calculations based on SPB model results in an L with a deviation of less than 10% as compared with a more rigorous single non-parabolic band and multiple band model calculations. It is well known that the L value is used to estimate the lattice thermal conductivity, which will not change the total thermal conductivity and final ZT values. The Lorenz number is given by the formula:

$$L=\left(\frac{k_{\mathrm{B}}}{e}\right)^{2}\left(\frac{(r+7/2)F_{r+5/2}(\eta)}{(r+3/2)F_{r+1/2}(\eta)}-\left[\frac{(r+5/2)F_{r+3/2}(\eta)}{(r+3/2)F_{r+1/2}(\eta)}\right]^{2}\right) \tag{S1}$$

where k_{B} is the Boltzmann constant and η represents the reduced Fermi energy, which can be derived from the measured Seebeck coefficients *via* the following equations:

$$S=\pm\frac{k_{\mathrm{B}}}{e}\left(\frac{(r+5/2)F_{r+3/2}(\eta)}{(r+3/2)F_{r+1/2}(\eta)}-\eta\right) \tag{S2}$$

where $F_n(\eta)$ is the nth order Fermi integral:

$$F_{\mathrm{n}}(\eta)=\int_{0}^{\infty}\frac{\chi^{\mathrm{n}}}{1+e^{\chi-\eta}}\,\mathrm{d}\chi \tag{S3}$$

$$\eta=\frac{E_{\mathrm{f}}}{k_{\mathrm{B}}T} \tag{S4}$$

4. Klemens-Drabble model

At a temperature above the Debye temperature Θ_D, the ratio of the lattice thermal conductivities including point defects and that of parent material can be expressed as the following equation:[3]

$$\frac{\kappa_{\mathrm{lat}}}{\kappa_{\mathrm{lat,\,p}}}=\frac{\tan^{-1}U}{U} \tag{S5}$$

where κ_{lat} and $\kappa_{\mathrm{lat,p}}$ are the lattice thermal conductivities of the defected and parent materials, respectively. U is defined as:

$$U=\left(\frac{\pi^{2}\Theta_{D}\Omega}{hv_{\mathrm{a}}^{2}}\kappa_{\mathrm{lat,p}}\Gamma\right)^{\frac{1}{2}} \tag{S6}$$

where Θ_D, Ω, h and v_a, represent the Debye temperature, average atom volume, Planck constant and average sound velocity, respectively. The data are taken from reference.[4] Γ, the imperfection scaling parameter is a weighted sum of the mass fluctuation Γ_M and strain field fluctuation Γ_S and can be written as: $\Gamma = \Gamma_M + \varepsilon\Gamma_S$, where ε is a phenomenological adjustable parameter related to the Poisson ratio (v_P) and Grüneisen parameter (γ).

Setting Pb alloying as an example. Γ is defined as:

$$\Gamma_{Sn_{1-x}Pb_xTe} = \frac{1}{2}\left(\frac{M_{(Sn,Pb)}}{\overline{M}}\right)^2 \Gamma_{(Sn,\,Pb)} \tag{S7}$$

$$\overline{M} = \frac{1}{2}\left(M_{Sn} + M_{Pb}\right) \tag{S8}$$

$$\Gamma_{(Sn,Pb)} = \Gamma_{M(Sn,Pb)} + \varepsilon\Gamma_{S(Sn,Pb)} \tag{S9}$$

$$\Gamma_{M(Sn,Pb)} = x(1-x)\left(\frac{\Delta M}{M_{(Sn,Pb)}}\right)^2 \tag{S10}$$

$$\Gamma_{S(Sn,Pb)} = x(1-x)\left(\frac{\Delta r}{r_{(Sn,Pb)}}\right)^2 \tag{S11}$$

where $M_{(Sn,Pb)} = (1-x)M_{Sn} + xM_{Pb}$, $\Delta M = M_{Pb} - M_{Sn}$ and $r_{(Sn,Pb)} = (1-x)r_{Sn} + xr_{Pb}$, $\Delta r = r_{Pb} - r_{Sn}$

Then we get:

$$\Gamma_{Sn_{1-x}Pb_xTe} = \frac{1}{2}x(1-x)\left(\frac{M_{(Sn,Pb)}}{\overline{M}}\right)^2\left[\left(\frac{\Delta M}{M_{(Sn,Pb)}}\right)^2 + \varepsilon\left(\frac{\Delta r}{r_{(Sn,Pb)}}\right)^2\right] \tag{S12}$$

Back to the equation S5, we can obtain the theoretical predication κ_{lat} with Klemens-Drabble model.

Same calculations can be applied to $SnTe_{1-x}S_x$ and $(SnTe)_{1-x}(PbS)_x$ alloys.

5. **Figures**

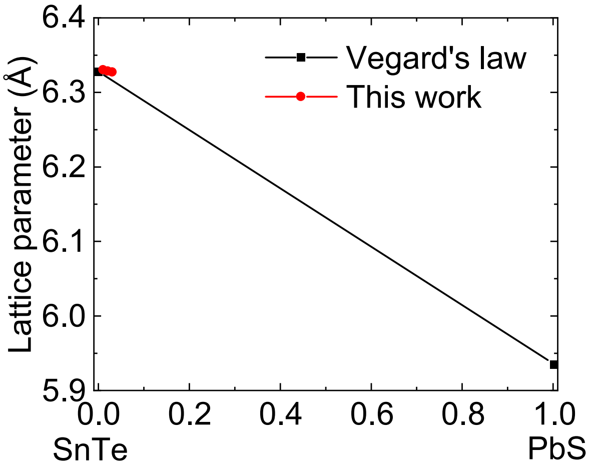

Figure S1. The lattice parameter as a function of PbS amount. Vegard's law is listed for comparison.

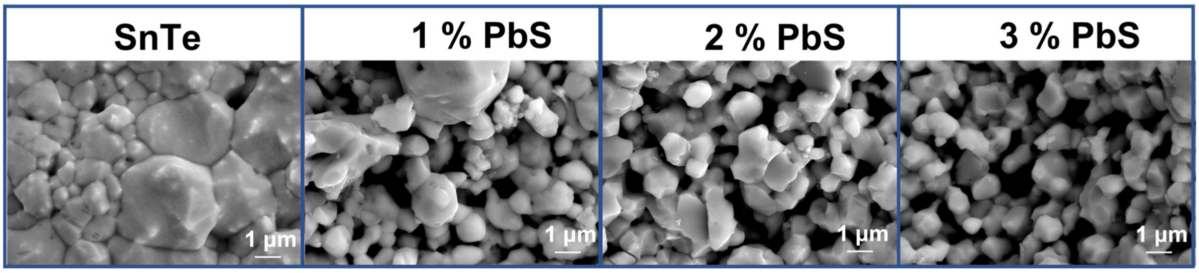

Figure S2. The SEM images of powders after annealing.

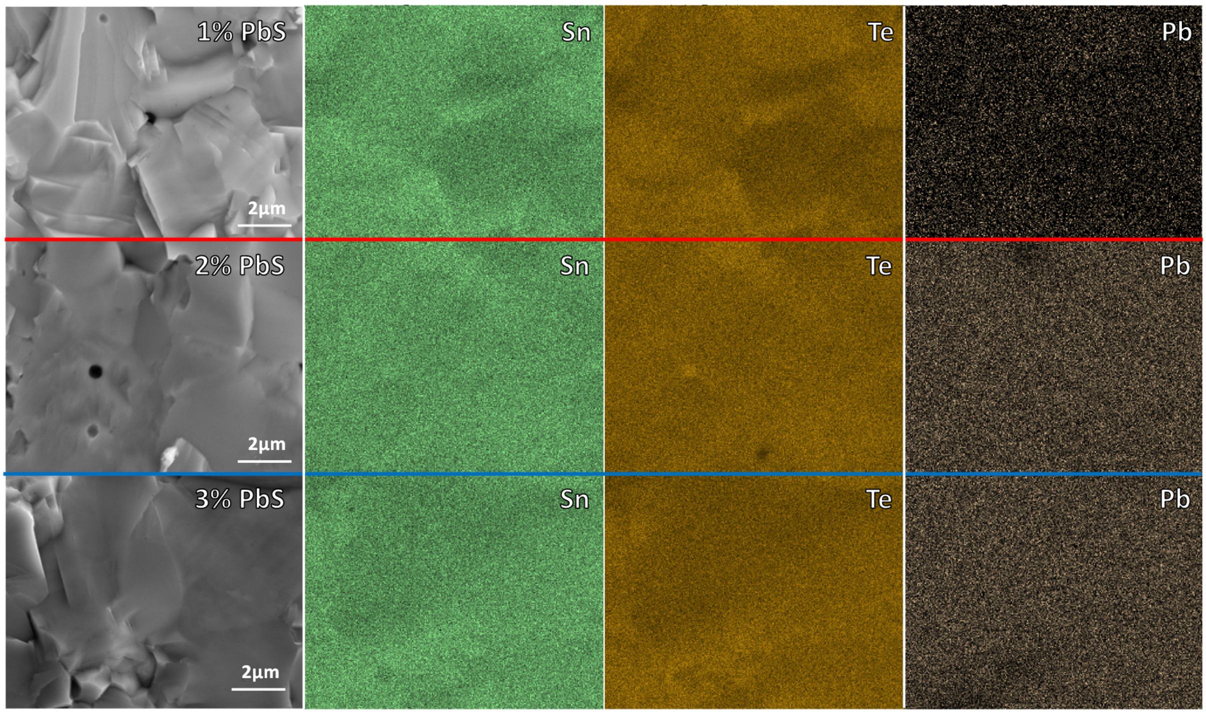

Figure S3. EDX mapping of SnTe-1/2/3% PbS.

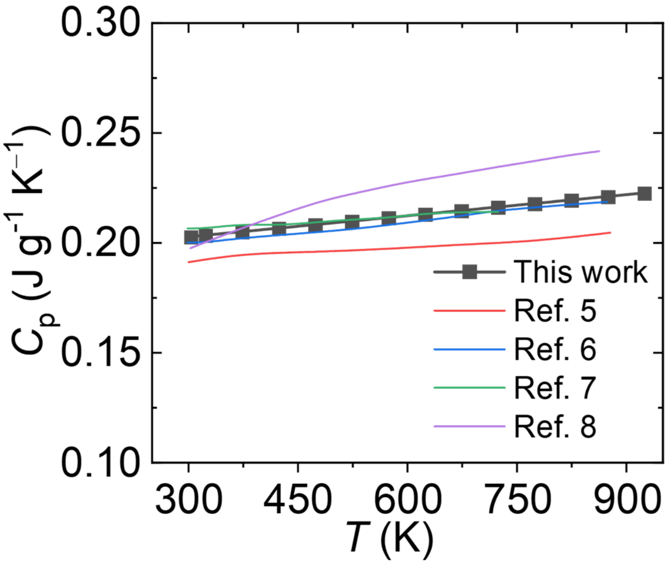

Figure S4. The heat capacity C_p of SnTe as a function of temperature. This figure of C_p values is taken from previous work by Zhao *et al.*[5] C_{p} in some other references are listed for comparision.[6-9]

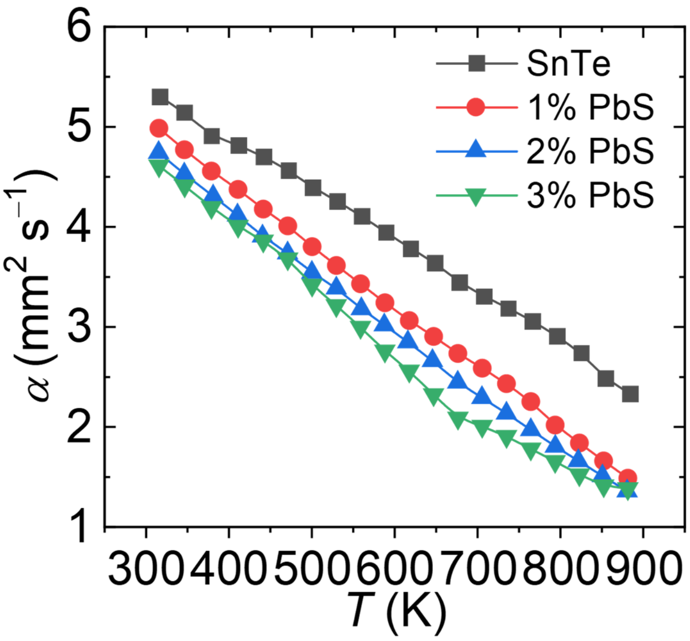

Figure S5. The temperature dependent thermal diffusivity of SnTe-xPbO nanocomposites (x=0, 1%, 2%, 3%).

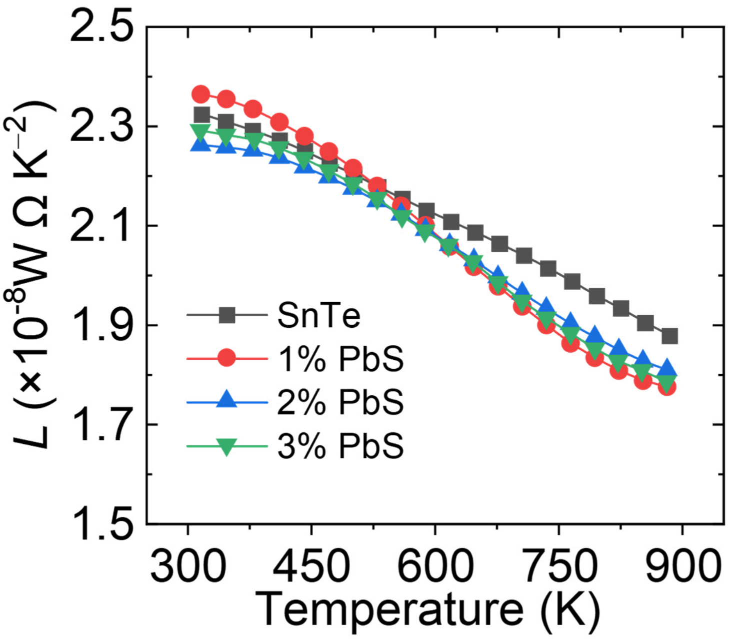

Figure S6. The temperature dependent Lorenz number of SnTe-xPbO nanocomposites (x=0, 1%, 2%, 3%).

6. Reference

1. Han, G.; Zhang, R.; Popuri, S. R.; Greer, H. F.; Reece, M. J.; Bos, J.-W. W. G.; Zhou, W.; Knox, A. R.; Gregory, D. H., Large-scale surfactant-free synthesis of p-Type SnTe nanoparticles for thermoelectric applications. *Materials* **2017,** 10, 233.
2. McCarthy, C. L.; Webber, D. H.; Schueller, E. C.; Brutchey, R. L., Solution-Phase Conversion of Bulk Metal Oxides to Metal Chalcogenides Using a Simple Thiol-Amine Solvent Mixture. *Angew. Chem., Int. Ed.* **2015,** 54, 8378-8381.
3. Pei, Y. L.; He, J. Q.; Li, J. F.; Li, F.; Liu, Q. J.; Pan, W.; Barreteau, C.; Berardan, D.; Dragoe, N.; Zhao, L. D., High thermoelectric performance of oxyselenides: intrinsically low thermal conductivity of Ca-doped BiCuSeO. *Npg Asia Materials* **2013,** 5.
4. Tan, G.; Shi, F.; Hao, S.; Chi, H.; Bailey, T. P.; Zhao, L. D.; Uher, C.; Wolverton, C.; Dravid, V. P.; Kanatzidis, M. G., Valence Band Modification and High Thermoelectric Performance in SnTe Heavily Alloyed with MnTe. In *J. Am. Chem. Soc.*, American Chemical Society: 2015; Vol. 137, pp 11507-11516.
5. Zhao, L. D.; Zhang, X.; Wu, H.; Tan, G.; Pei, Y.; Xiao, Y.; Chang, C.; Wu, D.; Chi, H.; Zheng, L.; Gong, S.; Uher, C.; He, J.; Kanatzidis, M. G., Enhanced Thermoelectric Properties in the Counter-Doped SnTe System with Strained Endotaxial SrTe. *J. Am. Chem. Soc.* **2016,** 138, 2366-2373.
6. Zhang, Q.; Liao, B.; Lan, Y.; Lukas, K.; Liu, W.; Esfarjani, K.; Opeil, C.; Broido, D.; Chen, G.; Ren, Z., High thermoelectric performance by resonant dopant indium in nanostructured SnTe. *Proc. Natl. Acad. Sci. U. S. A.* **2013,** 110, 13261-13266.
7. Pei, Y.; Zheng, L.; Li, W.; Lin, S.; Chen, Z.; Wang, Y.; Xu, X.; Yu, H.; Chen, Y.; Ge, B., Interstitial Point Defect Scattering Contributing to High Thermoelectric Performance in SnTe. *Advanced Electronic Materials* **2016,** 2, 1600019.
8. Banik, A.; Shenoy, U. S.; Anand, S.; Waghmare, U. V.; Biswas, K., Mg alloying in SnTe facilitates valence band convergence and optimizes thermoelectric properties. *Chem. Mater.* **2015,** 27, 581-587.
9. Zhou, M.; Gibbs, Z. M.; Wang, H.; Han, Y.; Xin, C.; Li, L.; Snyder, G. J., Optimization of thermoelectric efficiency in SnTe: The case for the light band. *Phys. Chem. Chem. Phys.* **2014,** 16, 20741-20748.

References in Figure 7

1. Liu, X.; Zhang, B.; Chen, Y.; Wu, H.; Wang, H.; Yang, M.; Wang, G.; Xu, J.; Zhou, X.; Han, G., Achieving Enhanced Thermoelectric Performance in (SnTe)1-x(Sb2Te3)x and (SnTe)1-y(Sb2Se3)y Synthesized via Solvothermal Reaction and Sintering. In *ACS Appl. Mater. Interfaces*, American Chemical Society (ACS): 2020; Vol. 12, pp 44805-44814.

2. Yang, B.; Li, S.; Li, X.; Wang, Y.; Zhong, H.; Feng, S., Microstructure and enhanced thermoelectric performance of Te–SnTe eutectic composites with self-assembled rod and lamellar morphology. In *Intermetallics*, Elsevier Ltd: 2019; Vol. 112, p 106499.

3. Han, G.; Zhang, R.; Popuri, S. R.; Greer, H. F.; Reece, M. J.; Bos, J.-W. W. G.; Zhou, W.; Knox, A. R.; Gregory, D. H., Large-scale surfactant-free synthesis of p-Type SnTe nanoparticles for thermoelectric applications. In *Materials*, 2017/08/05 ed.; MDPI AG: 2017; Vol. 10, p 233.

4. Lu, W.; He, T.; Li, S.; Zuo, X.; Zheng, Y.; Lou, X.; Zhang, J.; Li, D.; Liu, J.; Tang, G., Thermoelectric performance of nanostructured In/Pb codoped SnTe with band convergence and resonant level prepared: Via a green and facile hydrothermal method. In *Nanoscale*, Royal Society of Chemistry: 2020; Vol. 12, pp 5857-5865.

5. Xu, E. Z.; Li, Z.; Martinez, J. A.; Sinitsyn, N.; Htoon, H.; Li, N.; Swartzentruber, B.; Hollingsworth, J. A.; Wang, J.; Zhang, S. X., Diameter dependent thermoelectric properties of

individual SnTe nanowires. In *Nanoscale*, Royal Society of Chemistry: 2015; Vol. 7, pp 2869-2876.

6. Ibañ ez, M.; Hasler, R.; Genc, A.; Liu, Y.; Kuster, B.; Schuster, M.; Dobrozhan, O.; Cadavid, D.; Arbiol, J.; Cabot, A.; Kovalenko, M. V., Ligand-mediated band engineering in bottom-up assembled SnTe nanocomposites for thermoelectric energy conversion. In *J. Am. Chem. Soc.*, 2019/04/25 ed.; 2019; Vol. 141, pp 8025-8029.

7. Ju, H.; Kim, J., Anion-exchanged porous SnTe nanosheets for ultra-low thermal conductivity and high-performance thermoelectrics. In *Chem. Eng. J.*, Elsevier B.V.: 2020; Vol. 402, p 126274.

8. Wang, L.; Chang, S.; Zheng, S.; Fang, T.; Cui, W.; Bai, P. P.; Yue, L.; Chen, Z. G., Thermoelectric Performance of Se/Cd Codoped SnTe via Microwave Solvothermal Method. In *ACS Appl. Mater. Interfaces*, American Chemical Society: 2017; Vol. 9, pp 22612-22619.

9. Chen, W. H.; Yang, Z. R.; Lin, F. H.; Liu, C. J., Nanostructured SnSe: hydrothermal synthesis and disorder-induced enhancement of thermoelectric properties at medium temperatures. In *J. Mater. Sci.*, Springer New York LLC: 2017; Vol. 52, pp 9728-9738.

10. Li, Z.; Chen, Y.; Li, J. F.; Chen, H.; Wang, L.; Zheng, S.; Lu, G., Systhesizing SnTe nanocrystals leading to thermoelectric performance enhancement via an ultra-fast microwave hydrothermal method. In *Nano Energy*, Elsevier Ltd: 2016; Vol. 28, pp 78-86.

11. Feng, D.; Ge, Z. H.; Chen, Y. X.; Li, J.; He, J., Hydrothermal synthesis of SnQ (Q = Te, Se, S) and their thermoelectric properties. In *Nanotechnology*, 2017/10/19 ed.; 2017; Vol. 28, p 455707
